# Enhanced Adsorption of Gaseous Naphthalene by Activated Carbon Fibers at Elevated Temperatures

**DOI:** 10.3390/toxics12080537

**Published:** 2024-07-24

**Authors:** Chiou-Liang Lin, Chun-Yi Huang, Zhen-Shu Liu

**Affiliations:** 1 Department of Civil and Environmental Engineering, National University of Kaohsiung, Kaohsiung 811726, Taiwan; cllin0407@nuk.edu.tw; 2 Department of Safety, Health and Environmental Engineering, Ming Chi University of Technology, New Taipei City 24301, Taiwan; 3 Center for Sustainability and Energy Technologies, Chang Gung University, Taoyuan 33302, Taiwan; 4 Chronic Diseases and Health Promotion Research Center, Chang Gung University of Science and Technology, Chiayi 61363, Taiwan; 5 Biochemical Technology R&D Center, Ming Chi University of Technology, New Taipei City 24301, Taiwan

**Keywords:** activated carbon fibers (ACFs), adsorption, naphthalene, toluene, environmental pollution

## Abstract

This study utilized activated carbon fibers (ACFs) as adsorbents to investigate the removal efficiency of naphthalene and toluene at elevated temperatures and their competitive adsorption behavior. Three types of ACFs, inlet concentrations of naphthalene (343, 457, and 572 mg·Nm^−3^), and toluene (2055, 2877, and 4110 mg·Nm^−3^) were investigated to determine the adsorption capacities of naphthalene and toluene. To study the reaction mechanisms of naphthalene and toluene on the ACFs, the BET, SEM, FTIR, and TGA methods were used to examine the physical and chemical characteristics of ACFs. Results showed ACF-A’s superior adsorption capacity for naphthalene that was attributed to its mesoporous structure and hydrophobicity. Adsorption equilibrium studies indicated multilayer adsorption behavior. Competitive adsorption experiments demonstrated the displacement of toluene by naphthalene on ACF-A, highlighting its higher selectivity for naphthalene. Functional group analysis revealed changes in ACF surfaces after naphthalene adsorption, suggesting π-π dispersion and electron donor–acceptor interactions. Overall, this study underscores the importance of pore structure and surface properties in designing ACFs for the efficient adsorption of high-boiling-point organic pollutants.

## 1. Introduction

Organic compounds generated during the combustion process have led to severe air pollution and pose risks to human health. However, practical applications lack effective air pollution control devices for organic pollutants. Organic pollutants including volatile organic compounds (VOCs) and polycyclic aromatic carbons (PAHs) have attracted attention regarding their carcinogenic and toxic nature, with their concentrations being much lower than those of other pollutants. These organic compounds emitted into the atmosphere participate in photochemical reactions [1,2], leading to the rise in PM_2.5_ and ozone in the atmosphere. Therefore, devising an effective technique to remove organic compounds from flue gas is important and urgent to improve air quality.

Recovery methods (e.g., condensation, adsorption, absorption, and membrane separation) and destructive methods such as thermal incineration and catalytic oxidation have been developed to control organic compounds [1]. Among them, adsorption is the most widely used for the abatement of low-concentration organic pollutants [3,4]. For the achievement of high removal efficiency, different kinds of porous materials with a high specific surface area including activated carbon, activated carbon fiber, carbon nanotube, zeolite, metal organic framework, and silica gel are commonly used as adsorbents [1,5,6]. Of these adsorbents, activated carbon fibers (ACFs) have received considerable attention due to their much higher surface areas, uniform micropore structure, faster adsorption and desorption kinetics, and lower pressure drop [3,7,8,9,10,11,12].

The key factors affecting the adsorption capacities of VOCs and PAHs on carbonaceous adsorbents including the adsorption conditions (e.g., temperature, humidity, and flow velocity), properties of adsorbates (e.g., molecular structure, molecular polarity, and boiling point), as well as the physiochemical characters of adsorbents (e.g., specific surface area, pore size, and functional groups) have been investigated previously [3,7,13,14,15,16,17,18]. These studies determined that the adsorption capacities of VOCs and PAHs were predominantly influenced by the specific surface area and pore size of adsorbents. Liu et al. examined the adsorption characteristic of gaseous naphthalene on carbonaceous adsorbents. Their findings suggested that ordered mesoporous carbons with a narrower pore size range (2–5 nm) exhibited superior performance as sorbents for naphthalene adsorption [15]. Similar conclusions have been reported in other literature as well. Mo et al. indicated that mesoporous structures could capture larger organic molecules, thereby enhancing the adsorption of organic pollutants by activated carbon [16]. Hou et al. demonstrated that ordered mesoporous carbon (OMC) with a pore diameter of 4 nm exhibited the highest adsorption capacity for naphthalene [13]. A previous study also investigated the effects of five functional groups (carbonyl, hydroxyl, carboxyl, amino, and hydrogen groups) on the adsorption capacities of naphthalene, anthracene, and phenanthrene on activated carbon. The results revealed that the carbonyl functional group exhibited the most significant increase in the adsorption of PAHs compared to other functional groups [17]. Lin et al. demonstrated that the best adsorption capacities for toluene in ACFs were achieved through an optimal combination of a significant mesopore volume and low oxygen content [7].

As mentioned earlier, research on the adsorption characteristics of carbonaceous adsorbents has traditionally focused on volatile organic compounds (VOCs) at room temperature, with less attention paid to the adsorption and removal of gas-phase polycyclic aromatic hydrocarbons (PAHs), which have a high boiling point. Toluene and naphthalene can be found together in flue gases from industrial processes involving chemical manufacturing, petrochemical refining, and combustion activities such as incineration and vehicle exhaust. Their presence underscores the importance of monitoring and controlling emissions to minimize environmental and health risks. Activated carbon fibers, akin to conventional activated carbons but possessing a higher surface area and featuring mesopores and micropores, exhibit enhanced affinity for large molecular PAHs. Additionally, their woven or structured fabric configuration reduces typical pressure loss during adsorption processes and presents opportunities for the effective removal of particulate pollutants from smoke emissions. This study initiated an exploratory investigation into the adsorption efficiency of gaseous naphthalene at elevated temperatures. Simultaneously, it assessed whether the coexistence of gaseous toluene impacts the removal efficiency of gaseous naphthalene, aiming to evaluate their potential application in treating emissions from practical industrial flue gas.

## 2. Experimental Section

### 2.1. Preparation of ACFs

In this study, the three types of activated carbon fibers utilized were commercially produced by Taiwan Carbon Technology Co., Ltd. (TCT) (Taichung, Taiwan). The ACFs were derived from a polyacrylonitrile (PAN) precursor. Preceding the initiation of experiments, a requisite pre-treatment was conducted on the activated carbon fibers to eliminate potential impurities that could influence the outcomes of the adsorption experiments. Initially, the fibers were immersed in deionized water (DI water) for a 2 h cleaning duration at a rotational speed of 50 rpm. Following this, they were subjected to a 24 h drying period in an oven at 105 °C. Three weightings were conducted during this period to confirm the stable weight of the ACFs. Subsequently, they were cooled and stored in a brown container for preservation. Therefore, after the 24 h drying period, it can be ensured that all ACFs are completely free of moisture.

### 2.2. Characterization of ACFs

To investigate the impact of ACFs’ porosity on the competitive adsorption of naphthalene and toluene, the BET surface area analyzer (ASAP 2020, Micrometertics Instrument Corporation, Norcross, GA, USA) was employed to ascertain the porosities and BET surface area of the ACFs through N_2_ adsorption–desorption isotherms at 77 K. Prior to N_2_ sorption analysis, the samples underwent preheating to 200 °C for degassing and were subsequently cooled to room temperature under vacuum conditions. The micropore volumes (pores with sizes < 2 nm) of the ACFs were determined using the t-Plot method. Mesopore volumes (pores with sizes ranging from 2 to 50 nm) were calculated by subtracting the micropore volumes (determined via the t-Plot method) from the total pore volume at a relative pressure of 0.99.

The morphology of three ACFs was examined through field emission scanning electron microscopy (FE-SEM; Model JSM-6700F, JEOL, Tokyo, Japan) operated at 5 kV. The thermal stability of the three ACFs was assessed using a thermo-gravimetric analyzer (TGA; STA6000, PerkinElmer, Shelton, CT, USA). Surface functional groups of the ACFs were characterized using a Fourier-transform infrared spectrometer (FTIR; Spectrum One, PerkinElmer, Shelton, CT, USA). Infrared spectra were recorded in the range of 450 to 4000 cm^−1^. The FTIR analysis was conducted after grinding the ACFs and blending them with KBr powder to create sample KBr pellets, with an approximate ACFs-to-KBr ratio of 1/50.

### 2.3. Dynamic Adsorption of Naphthalene and Toluene

**Figure 1:**
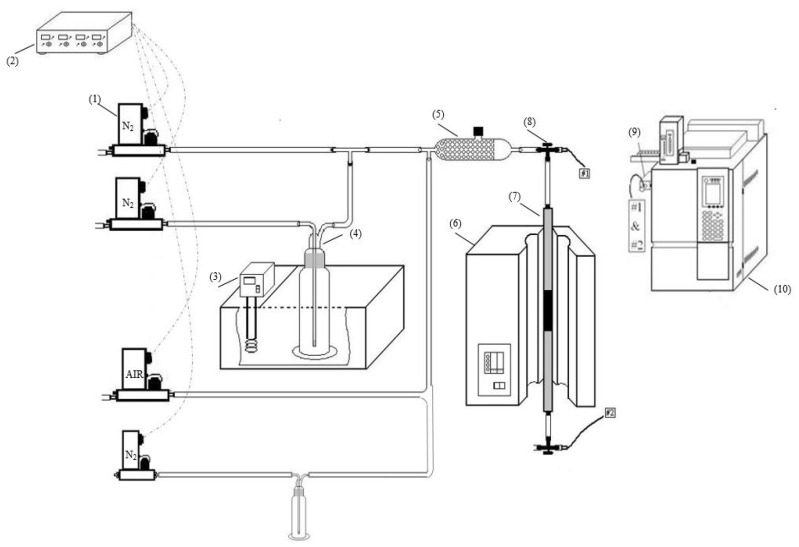
Experimental setup: (1) mass flow controller, MFC, (2) power supply of MFC readout, (3) thermostat water bath, (4) gas bubbler filled with naphthalene or toluene, (5) gas mixing chamber, (6) heating unit, (7) adsorption bed, (8) three-way valve, (9) gas sampling, (10) GC–FID [7].

The adsorption of naphthalene and toluene onto the ACFs was conducted within a fixed-bed reactor. To load the fibers into the fixed-bed reactor, the ACFs were cut into circular shapes with a diameter of 10 mm and then secured in place using steel wire and a steel net. The experimental arrangement employed in this study is illustrated in Figure 1. This setup can be delineated into three main sections: (1) preparatory steps for naphthalene and toluene, (2) the adsorption process, and (3) the subsequent analysis of naphthalene and toluene. Gaseous naphthalene and toluene were generated by bubbling nitrogen into solid naphthalene tablets and liquid toluene, respectively, both maintained at 303 K in a thermostat water bath. Subsequently, the resulting gaseous naphthalene and toluene were mixed with nitrogen and air sourced from two steel cylinders in a gas mixing chamber to achieve the desired concentrations of gaseous naphthalene, toluene, and oxygen. Once the inlet concentrations of gaseous naphthalene, toluene, and oxygen stabilized, the gases were introduced into the fixed-bed reactor. The concentrations of gaseous naphthalene and toluene at the inlet and outlet of the reactor were continuously monitored using an online gas chromatograph (GC-2014, Shimadzu Corporation, Kyoto, Japan). The operational parameters included three types of ACFs, inlet concentrations of naphthalene (343, 457, and 572 mg·Nm^−3^), and inlet concentrations of toluene (2055, 2877, and 4110 mg·Nm^−3^). The adsorption temperature was maintained at 70 °C. The dynamic adsorption capacity was calculated using the following Equation (1):

(1) $$q=\frac{Q\int_{0}^{t}\left(C_{in}-C_{out}\right)dt}{m}$$

where *q* (mg·g^−1^) is the adsorption capacity of naphthalene or toluene; *Q* (Nm^3^·min^−1^) is the gas flow rate; *m* (g) is the mass of adsorbent; and *C_in_* (mg·Nm^−3^) and *C_out_* (mg·Nm^−3^) are the concentrations of naphthalene or toluene at the inlet and outlet, respectively. *t* represents the adsorption equilibrium time (min).

### 2.4. Adsorption Equilibrium Model

To study the adsorption equilibrium of naphthalene on the ACFs, experiments were also conducted with different concentrations of naphthalene on ACF-A. Langmuir and Freundlich adsorption isothermal equations were employed to match the adsorption data. Langmuir and Freundlich equations are presented in Equations (2) and (3) [10,19,20]. *C_e_* was calculated using the following Equation (4):

(2) $$q_{e}=\frac{q_{m}k_{L}C_{e}}{1+k_{L}C_{e}}$$

(3) $$q_{e}=k_{F}{C_{e}}^{\frac{1}{n}}$$

(4) $$C_{e}=\frac{Q\int_{0}^{t}C_{out}dt}{Q\times t}$$

where *C_e_* (mg·Nm^−3^), *q_e_* (mg·g^−1^), and *q_m_* (mg·g^−1^) are the equilibrium concentration, equilibrium adsorption capacity, and maximum adsorption capacity, respectively. *k_L_* (Nm^3^·mg^−1^) is the equilibrium adsorption constant of the Langmuir model. *k_F_* ((mg·g^−1^)(Nm^3^·mg^−1^)^1/*n*^) and *n* are the equilibrium adsorption constant and adsorption intensity of the Freundlich model, respectively. *Q* (Nm^3^·min^−1^) is the gas flow rate; *C_out_* (mg·Nm^−3^) is the concentration of naphthalene or toluene at the outlet; and *t* represents the adsorption equilibrium time (min).

## 3. Results and Discussion

### 3.1. Characterization of the ACFs

**Figure 2:**
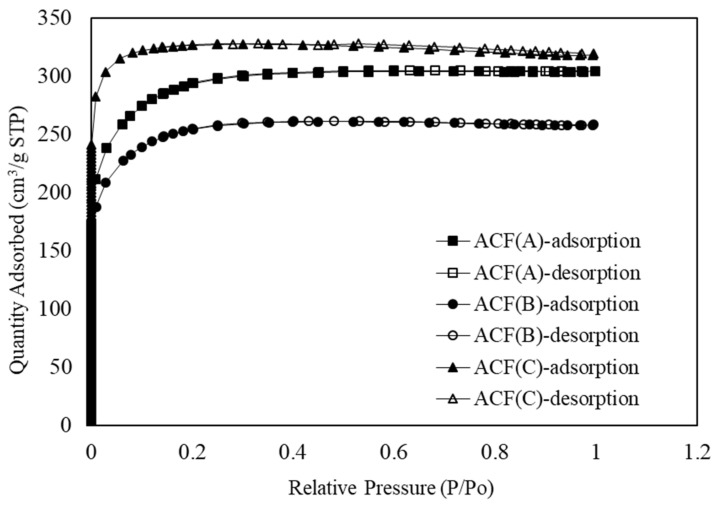
Nitrogen adsorption–desorption isotherm of ACF-A, ACF-B, and ACF-C.

Figure 2 illustrates the nitrogen adsorption/desorption isotherms of the three types of ACFs. According to the IUPAC definition, the adsorption isotherms of all three ACFs fall into the category of Type I, indicating the characteristic adsorption behavior of typical microporous materials. The results reveal that the maximum adsorption of the three ACFs was achieved at a relative pressure (P/P_0_) approaching 0.2. As the relative pressure gradually increased to the range of 0.3 to 0.4, the nitrogen adsorption tended to level off, signifying narrow pore adsorption. Furthermore, the adsorption quantity was predominantly controlled by the micropore volume. Additionally, ACF-C exhibited hysteresis in the adsorption/desorption isotherm, characterized by a Type H4 isotherm. This indicated the presence of fine, narrow slit-like pores in the structure of ACF-C [21].

Table 1 presents the porous structure characteristics and specific surface area for the three types of ACFs. The results indicate that the specific surface area of the three ACFs ranged between 800 and 1100 m^2^/g, with pore volumes falling within the range of 0.4 to 0.5 cm^3^/g and average pore diameters of approximately 1.8 nm. Additionally, the order of the specific surface area, total pore volume, and micropore volume among the three ACFs is ACF-C > ACF-A > ACF-B. Meanwhile, the mesopore volume follows the sequence ACF-A > ACF-B > ACF-C, corresponding to the order of their naphthalene adsorption capacity. Notably, ACF-C exhibited a predominant presence of micropores, constituting 94% of the total pore volume.

**Figure 3:**
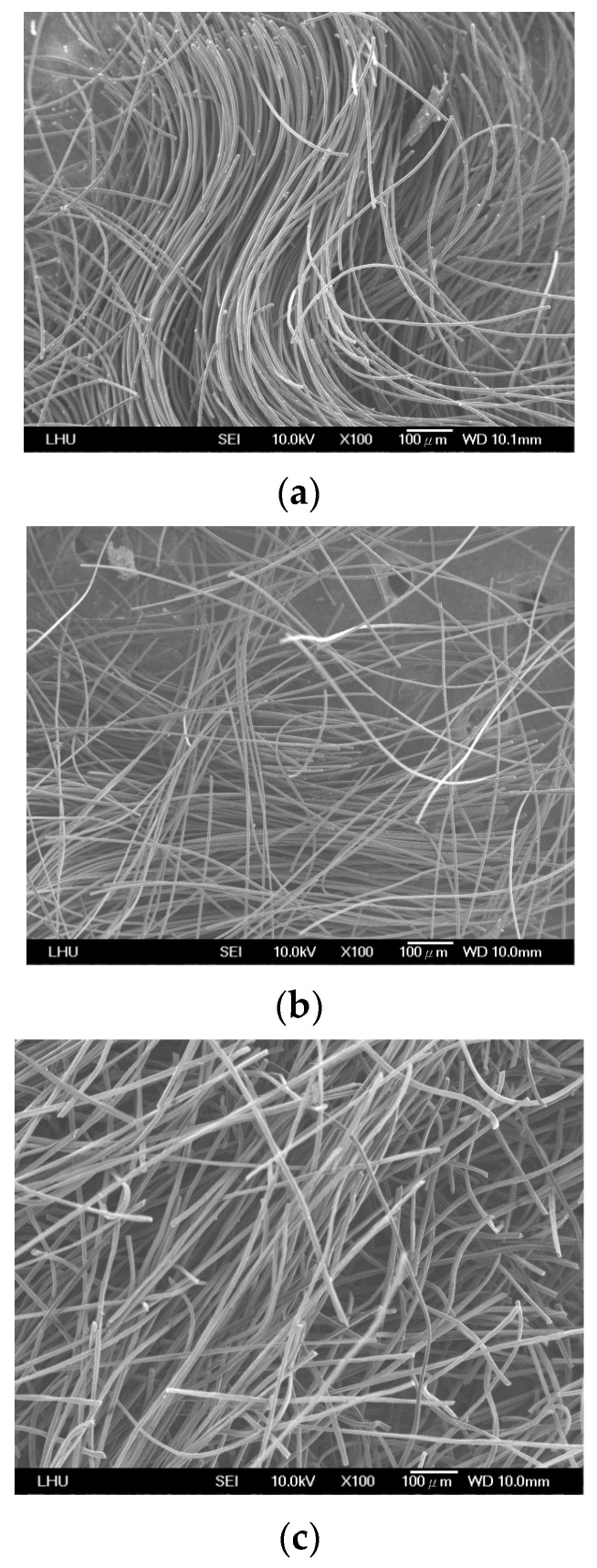
The SEM micrographs of ACFs: (**a**) ACF-A, (**b**) ACF-B, (**c**) ACF-C.

Figure 3 shows the structural characteristics of the three ACFs, revealing a filamentous arrangement in all ACFs. ACF-B and ACF-C exhibited a more disorderly alignment, while ACF-A displayed a comparatively orderly and uniform structure. Because ACF-A had a tighter weave structure enabling adequate gas contact, it assisted in enhancing the adsorption of naphthalene. The adsorption results for naphthalene (Run 1~Run 3) across the three types of ACFs demonstrate an adsorption capacity order of ACF-A > ACF-B > ACF-C. The tighter packing of fibers in ACF-A, compared to ACF-B and ACF-C, facilitated enhanced gas capture and interaction, potentially contributing to the heightened adsorption capacity for naphthalene.

**Figure 4:**
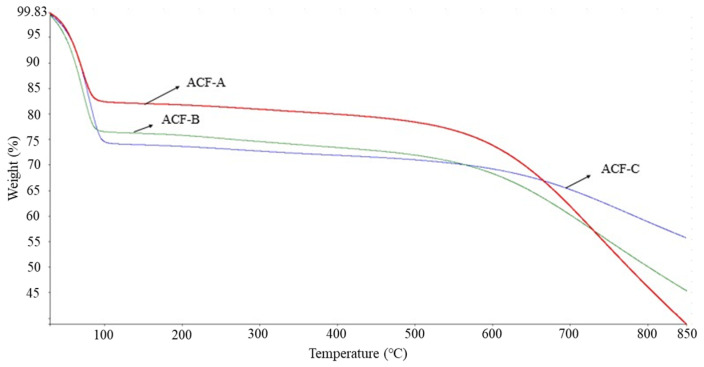
Thermal stability of various ACFs with TGA analysis.

Figure 4 illustrates the thermal stability of the three ACFs. It can be observed that all three ACFs experienced a first-stage weight loss between 29 and 100 °C, corresponding to the moisture absorbed by the ACFs. The weight loss for ACF-A was approximately 17%, while ACF-B and ACF-C exhibited losses of around 24% and 26%, respectively. This suggests that ACF-A possesses a more pronounced hydrophobic nature compared to ACF-B and ACF-C. The adsorption results for the three ACFs (Run 1 to Run 3) reveal a sequence in naphthalene adsorption capacity as follows: ACF-A > ACF-B > ACF-C, which correlates with the hydrophobicity of the ACFs. As the temperature continued to rise to approximately 550 °C, a second-stage weight loss occurred, indicating the onset of structural degradation in the ACFs. Consequently, the results suggest that the thermal stability of ACFs can be sustained up to around 500 °C.

### 3.2. Effects of ACF Characteristics on Naphthalene Adsorption

**Figure 5:**
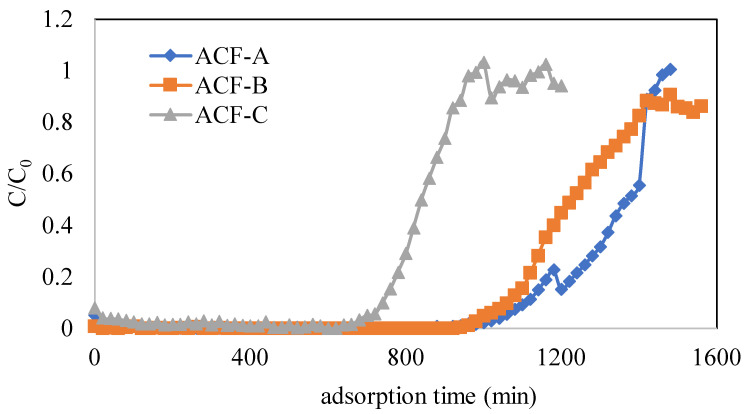
Adsorption breakthrough curves of naphthalene for different ACFs (Runs 1–3; naphthalene concentration = 572 mg·Nm^−3^, toluene concentration = 0 ppm).

Detailed operational conditions and adsorption capacities for various tests are presented in Table 2. The operational parameters included three types of ACFs (Runs 1–3), inlet concentrations of naphthalene (343, 457, and 572 mg·Nm^−3^) (Run 1 and Runs 4–5), and inlet concentrations of toluene (2055, 2877, and 4110 mg·Nm^−3^) (Runs 7–9). To investigate the impact of three types of ACFs on naphthalene adsorption, experiments were conducted at a naphthalene concentration of 572 mg·Nm^−3^ (as detailed in Runs 1–3 in Table 2). Figure 5 illustrates the adsorption breakthrough curves of naphthalene for different ACFs. The breakthrough times for ACF-A, ACF-B, and ACF-C were 1060, 1020, and 700 min, respectively. The saturated adsorption capacities of ACF-A, ACF-B, and ACF-C were calculated using Equation (1) and found to be 493, 401, and 345 mg/g (as shown in Table 2), respectively. These findings substantiate that the sequence of adsorption capacity for naphthalene among the three activated carbon fibers is ACF-A > ACF-B > ACF-C, in accordance with the mesopore volumes (as shown in Table 1) and hydrophobic characteristics (as shown in Figure 4) of the ACFs. Despite ACF-C possessing the highest specific surface area, it exhibited the lowest adsorption capacity for naphthalene due to the absence of mesopore volume. This observation highlights that the pore size distribution of the adsorbent has a more profound impact on the adsorption performance for naphthalene than the specific surface area of the adsorbent.

The ratio of adsorbent pore size to adsorbate molecule size is a key factor influencing the adsorption performance of the adsorbent. Optimal adsorption performance is generally achieved when the size ratio falls within the range of 1.7 to 3.0 [1,13,14]. Given the molecule size of naphthalene being 6.777 Å × 4.995 Å, the optimal pore size range for adsorbents designed for naphthalene adsorption is estimated to be between 1 and 2 nm. Adsorbents exclusively comprised of micropores may impede the diffusion rate of naphthalene within the pores, resulting in a reduction in their adsorption capacity [13]. Hou et al. indicated that ordered porous silica with a pore diameter of 2.3 nm exhibited the maximum adsorption capacity for naphthalene [14]. The present study provides evidence that hydrophobic adsorbents with a combination of microporous and mesoporous structures are beneficial for adsorbing large molecules of nonpolar organic compounds, such as naphthalene.

### 3.3. Adsorption Equilibrium of Naphthalene

**Figure 6:**
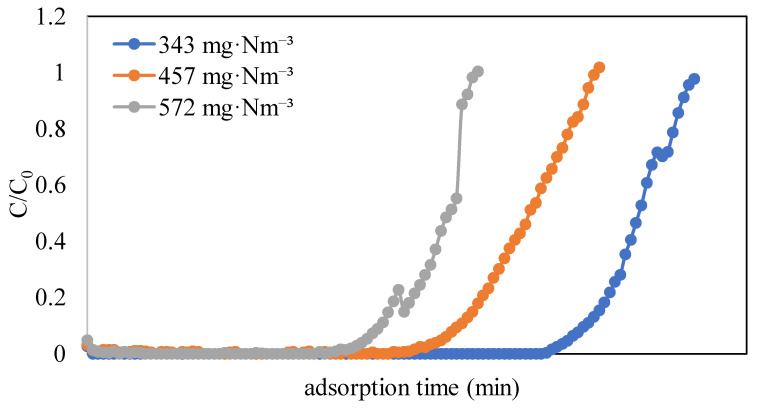
Adsorption breakthrough curves of naphthalene on ACF-A at different naphthalene concentrations (Run 1 and Runs 4–5; toluene concentration = 0 ppm).

**Figure 7:**
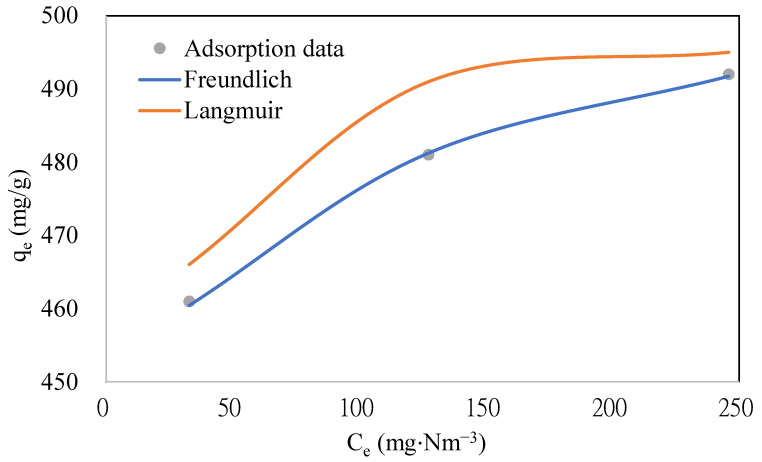
The adsorption equilibrium models for naphthalene on ACF-A (Run 1 and Runs 4–5; toluene concentration = 0 ppm).

To study the adsorption equilibrium of naphthalene on the ACFs, experiments were conducted with three concentrations of naphthalene (343, 457, and 572 mg·Nm^−3^) on ACF-A (as detailed in Run 1 and Runs 4–5 in Table 2). The adsorption breakthrough curves of naphthalene on ACF-A, depicted in Figure 6, reveal that breakthrough times were 1840 min, 1360 min, and 1060 min for concentrations of 343 mg·Nm^−3^, 457 mg·Nm^−3^, and 572 mg·Nm^−3^, respectively. The breakthrough time demonstrated a decreasing trend as the concentration increased. Correspondingly, the saturated adsorption capacities were 461 mg/g, 481 mg/g, and 493 mg/g (as shown in Table 2), indicating an increase in saturated adsorption capacity with elevated naphthalene concentrations. Figure 7 shows the adsorption equilibrium models for naphthalene on ACF-A. The equilibrium constant and saturated adsorption capacity are listed in Table 3. As illustrated in Figure 7, the adsorption capacity exhibits a uniform increase within the concentration range of 343–572 mg·Nm^−3^ (60–100 ppmv) for naphthalene, and saturation has not been reached. In accordance with the BDDT (Brunauer, Deming, Deming, and Teller) adsorption isotherm classification [22], the adsorption isotherm of naphthalene on ACF-A is classified as Type-II. The more suitable description for the adsorption equilibrium of naphthalene on ACF-A is provided by the Freundlich isothermal model (R^2^ > 0.99), suggesting the existence of multilayer adsorption or non-homogeneous adsorption equilibria.

### 3.4. Competitive Adsorption of Gaseous Naphthalene and Toluene

**Figure 8:**
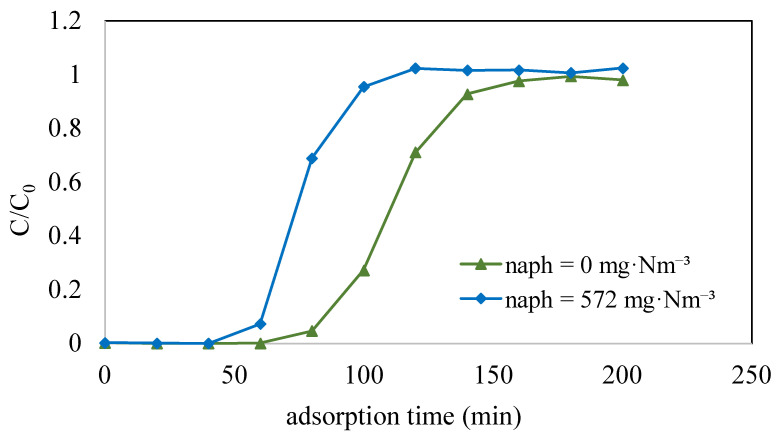
Adsorption breakthrough curves of toluene on ACF-A both in the presence and absence of naphthalene (Run 6 and Run 9; toluene concentration = 4110 mg·Nm^−3^).

This study initially focused on determining the adsorption capacity of toluene when it was present in isolation, aiming to assess whether the existence of naphthalene affected the adsorption capacity of toluene on ACF-A through competitive adsorption (as detailed in Run 6 and Run 9 in Table 2). Figure 8 illustrates the adsorption breakthrough curves of toluene on ACF-A both in the presence and absence of naphthalene. The results indicate that in the absence of naphthalene, toluene exhibited a breakthrough time of 80 min, with a saturation adsorption capacity of 293 mg/g. Conversely, in the presence of both toluene (concentration = 4110 mg·Nm^−3^) and naphthalene (concentration = 572 mg·Nm^−3^), the breakthrough curve for toluene indicated a shortened breakthrough time of 60 min, accompanied by a reduction in the saturation adsorption capacity to 213 mg/g. In binary-component co-adsorption, the adsorption capacity of toluene was found to decrease compared to its capacity in single-component adsorption, providing evidence of competitive adsorption between toluene and naphthalene.

**Figure 9:**
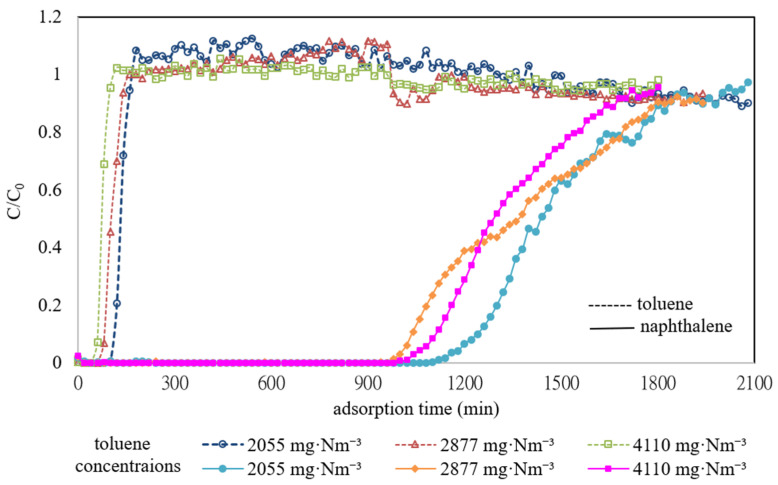
Adsorption breakthrough curves of toluene and naphthalene on ACF-A in the binary-component co-adsorption at different toluene concentrations (Runs 7–9; naphthalene concentration = 572 mg·Nm^−3^).

To further understand the competitive adsorption mechanism between toluene and naphthalene on ACF-A, binary-component co-adsorption experiments were conducted at different toluene concentrations (2055, 2877, and 4110 mg·Nm^−3^) while maintaining a naphthalene concentration of 572 mg·Nm^−3^ on ACF-A (as detailed in Runs 7–9 in Table 2). Figure 9 illustrates the breakthrough curves of toluene and naphthalene during these experiments. The findings reveal that in the presence of both toluene and naphthalene, the breakthrough time for naphthalene notably exceeds that for toluene. However, variations in toluene concentrations (2055, 2877, 4110 mg·Nm^−3^) did not significantly affect the breakthrough time for either toluene or naphthalene. Moreover, the breakthrough curve of toluene clearly indicates that once toluene reached adsorption saturation, its outlet concentration surpassed its inlet concentration, confirming the displacement of toluene molecules adsorbed on ACF-A by naphthalene molecules, leading to their subsequent desorption. Subsequently, as naphthalene gradually reached adsorption saturation, the outlet concentration of toluene fell below its inlet concentration, indicating renewed adsorption of toluene molecules by ACF-A. Table 2 demonstrates that in the presence of both naphthalene and toluene, the saturation adsorption capacity of naphthalene is notably higher than that of toluene. Additionally, when naphthalene and toluene coexist, the saturation adsorption capacities of both compounds are lower compared to when they are present individually. These results elucidate ACF-A’s higher adsorption selectivity for naphthalene over toluene. Applying both the Langmuir and Freundlich isothermal models to elucidate the adsorption equilibrium of toluene on ACF-A in binary-component co-adsorption, the finding (as shown in Table 3) validated that neither model was appropriate for characterizing the adsorption equilibrium of toluene (R^2^ < 0.99).

Hou et al. investigated the adsorption of gas-phase toluene and naphthalene on ordered mesoporous silica. The results confirmed that the pore size (2.3 nm) corresponding to the maximum adsorption capacity of naphthalene was larger than that of toluene (1.3 nm). Furthermore, naphthalene exhibited higher adsorption stability than toluene, demonstrating that naphthalene molecules could replace toluene molecules already adsorbed on the ordered mesoporous silica [14]. It is generally recognized that adsorption capacity is enhanced when the size ratio between adsorbent pores and adsorbate molecules ranges from 1.7 to 3.0. Table 1 highlights ACF-A as an adsorbent characterized by the coexistence of mesopores and micropores. Given the molecular sizes of naphthalene (6.777 Å × 4.995 Å) and toluene (5.435 Å × 4.313 Å), the size ratio of ACF-A’s pores to naphthalene molecules approaches the 1.7 to 3.0 range. Consequently, ACF-A exhibits an enhanced adsorption capacity towards naphthalene.

In recent years, the widespread application of carbonaceous adsorbents has spurred continuous efforts in scholarly literature to enhance their efficacy in removing volatile organic compounds (VOCs). Table 4 compiles the adsorption capacities of naphthalene and toluene on various carbon adsorbents, facilitating a comparison with the findings of this study. The table indicates that at ambient temperature (25 °C), adsorption capacities for naphthalene and toluene generally exceed those observed at elevated temperatures (125 °C). Modified microporous activated carbons typically exhibit higher adsorption capacities for toluene compared to their unmodified counterparts. Conversely, mesoporous carbon adsorbents enhance naphthalene adsorption while demonstrating less favorable adsorption of toluene. This study found optimal adsorption capacities of 493 mg/g for naphthalene and 293 mg/g for toluene. Although direct comparisons with the existing literature are hindered by varying experimental conditions, this research highlights the competitive performance of the tested activated carbon fibers (ACFs) in adsorption efficiency relative to mesoporous carbon adsorbents, suggesting promising potential for practical applications.

### 3.5. The Changes in Functional Groups on ACFs before and after Adsorption of Naphthalene

**Figure 10:**
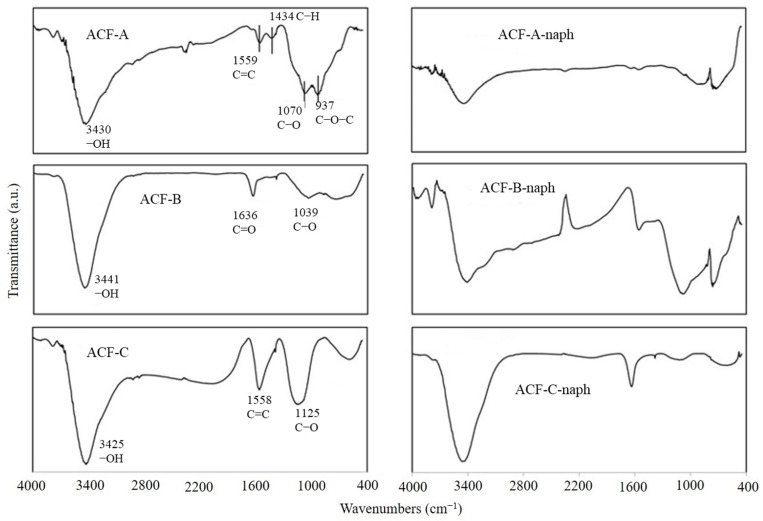
The changes in FTIR spectra of the ACF-A, ACF-B, ACF-C before and after the adsorption of naphthalene.

In addition to the pore size of ACFs affecting their adsorption efficiency towards naphthalene and toluene, the functional groups on the surface of ACFs may also play a significant role in influencing their adsorption efficiency for these compounds. To explore the effects of three types of ACFs on naphthalene adsorption, experiments were conducted at a naphthalene concentration of 572 mg·Nm^−3^ (as detailed in Runs 1–3 in Table 2). Figure 10 illustrates the FTIR spectra of ACF-A, ACF-B, and ACF-C before and after naphthalene adsorption. The results reveal that prior to adsorption, all three types of activated carbon fibers exhibited prominent and broad peaks at 3670–3000 cm^−1^, attributed to the vibration of O−H groups on the material surface or within the internal structure, or due to water adsorption [24]. Combined with the TGA results of this study (as shown in Figure 4), it is indicated that all three types of ACFs experienced a weight loss ranging from 15% to 25% between 29 and 100 °C, confirming that the peak at 3670–3000 cm^−1^ was caused by water adsorption. Additionally, ACF-A exhibited peaks at 1559, 1434, 1070, and 937 cm^−1^, representing the stretching vibration of C=C in aromatic compounds, the deformation vibration of C−H in single bond C(CH_3_)_3_, and the functional groups of C–O and C–O–C (esters, ethers, or phenols). ACF-B showed peaks at 1636 and 1039 cm^−1^, corresponding to the stretching vibration of C=O in ketones and C–O (ether) functional groups. ACF-C displayed peaks at 1558 and 1125 cm^−1^, indicating the stretching vibration of C=C in aromatic compounds and C–O (ether) functional groups [24,25,26].

Figure 10 also presents the changes in functional group positions following the adsorption of naphthalene on ACF-A, ACF-B, and ACF-C. With the exception of the O−H (3670–3000 cm^−1^) band, which remained unchanged due to water adsorption, other functional groups either disappeared or shifted positions, with ACF-B even exhibiting the emergence of new functional groups. Prior studies have explored the adsorption mechanisms of oxygen-containing functional groups on adsorbent surfaces and their interactions with organic compounds in aqueous environments, identifying two primary types of interactions between adsorbates and carbonaceous adsorbents: electrostatic and dispersive effects. The former arises only when organic compounds dissociate in water, whereas the latter encompasses three distinct mechanisms: hydrogen bonding formation, π-π dispersion, and electron donor–acceptor interactions [27,28,29]. Carbonaceous adsorbents featuring functional groups such as COOH, −OH, and −NH_2_ form hydrogen bonds with adsorbates [30]. However, the strength of hydrogen bond interactions between water molecules and functional groups surpasses those between aromatic compounds and surface functional groups on adsorbents, diminishing the likelihood of hydrogen bond interactions between aromatic compounds and adsorbents [31]. Pei et al. investigated the adsorption of 1,2,4-trichlorobenzene (TCB), 2,4,6-trichlorophenol (TCP), 2-naphthol, and naphthalene (NAPH) in liquid phase by graphene and graphene oxide. FTIR spectra confirmed that upon adsorption of TCB and TCP onto graphene, the peaks corresponding to the C=C stretching vibration and C−C skeletal vibrations of benzene rings experienced shifts, indicating the adsorption of TCB and TCP onto graphene via π-π interactions [32]. Considering the aforementioned findings, it is inferred that the reaction mechanism between naphthalene molecules and ACFs in this study may involve π-π dispersion and electron donor–acceptor interactions.

## 4. Conclusions

In conclusion, this study provides valuable insights into the adsorption of naphthalene using activated carbon fibers (ACFs) at elevated temperatures, addressing the scarcity of research in this area. The results demonstrate that ACF-A exhibits superior adsorption capacity for naphthalene compared to ACF-B and ACF-C, attributed to its higher mesopore volume and hydrophobic nature. Furthermore, the study reveals that the competitive adsorption of naphthalene and toluene on ACF-A leads to a decrease in the adsorption capacity of both compounds, highlighting the higher selectivity of ACF-A towards naphthalene over toluene. Additionally, the changes in functional groups on ACFs before and after the adsorption of naphthalene suggest the involvement of π-π dispersion and electron donor–acceptor interactions in the adsorption mechanism. These findings underscore the importance of considering the pore size distribution, surface characteristics, and competitive adsorption behavior when designing ACFs for the efficient removal of organic pollutants from gas streams. Overall, the study contributes valuable insights into optimizing ACFs for environmental remediation applications, particularly in industries where high-temperature flue gas containing naphthalene is prevalent. The findings of this study can provide insights into the removal of organic compounds during the combustion process in real-world industrial settings.

## Figures and Tables

**Table 1:** Specific surface area and pore volume of ACFs.

Sample	S_BET_ ^a^ (m^2^/g)	V_T_ ^b^ (cm^3^/g)	V_meso_ ^c^ (cm^3^/g)	V_micro_ ^d^ (cm^3^/g)	d_p_ ^e^ (nm)
ACF-A	1013	0.47	0.2	0.27	1.86
ACF-B	874	0.40	0.15	0.25	1.83
ACF-C	1098	0.49	0.03	0.46	1.79

^a^ Specific surface area by BET method; ^b^ total pore volume at P/P_0_ = 0.99; ^c^ (total pore volume) − (t-Plot micropore volume); ^d^ t-Plot micropore volume; ^e^ adsorption average pore width (4V/A by BET).

**Table 2:** Operational conditions and adsorption capacities of various tests.

	ACFs Type	Naphthalene Concentration (mg·Nm^−3^)	Toluene Concentration (mg·Nm^−3^)	Adsorption Temperature (°C)	Saturated Adsorption Capacity (mg/g)	
					Naphthalene	Toluene
Run 1	ACF-A	572	0	70	493	–
Run 2	ACF-B				401	–
Run 3	ACF-C				345	–
Run 4	ACF-A	343			461	–
Run 5		457			481	–
Run 6		0	4110		–	293
Run 7		572	2055		469	176
Run 8			2877		446	190
Run 9			4110		450	213

Other experimental conditions: oxygen content = 9.2%, retention time: 0.18 s, gas flow rate: 5 × 10^−4^ Nm^3^·min^−1^.

**Table 3:** Fitting parameters of adsorption equilibrium with Langmuir and Freundlich.

	Langmuir Isotherm			Freundlich Isotherm		
	*q_m_* (mg·g^−1^)	*k_L_* (m^3^·mg^−1^)	R^2^	*k_F_* (mg·g^−1^)(m^3^·mg^−1^)^1/*n*^	*n*	R^2^
Naphthalene (Run 1 and Runs 4–5)	500	0.417	0.952	410.92	30.67	0.998
Toluene (Runs 7–9)	224	0.0043	0.910	64.85	6.76	0.973

**Table 4:** Summary of the adsorption capacity of organic compounds on carbonaceous material.

Adsorbate	Adsorbent	Adsorption Capacity (mg g^−1^)	Experimental Condition	Reference
(1) naphthalene (2) toluene (3) binary-component	ordered mesoporous carbon (OMC)	(1) Q_nap_ = 1284 (2) Q_tol_ = 227 (3) Q_nap_ = 1210, Q_tol_ = 8	naph conc. = 200 mg⋅m^−3^, tol conc. = 400 mg⋅m^−3^, 25 °C, RH < 1%	[13]
naphthalene	(1) ordered mesoporous carbons (CMK-5) (2) carbon nanotubes (SWCNT) (3)activated carbon	(1) Q_nap_ = 222 (2) Q_nap_ = 113 (3) Q_nap_ = 190	naph conc. = 1024 mg⋅m^−3^, 125 °C	[15]
toluene	KOH-porous graphitized carbon fibers (PGCFs)	Q_tol_ = 662	tol conc. = 3760 mg⋅m^−3^, 25 °C	[12]
toluene	(1) porous graphitized carbon (PGC) (2) activated carbon	(1) Q_tol_ = 426 (2) Q_tol_ = 169	tol conc. = 3760 mg⋅m^−3^, 25 °C	[23]

## Data Availability

Dataset available on request from the authors.
